# Peripapillary Retinoschisis in Glaucoma Patients

**DOI:** 10.1155/2016/1612720

**Published:** 2016-03-16

**Authors:** Serife Bayraktar, Zafer Cebeci, Melis Kabaalioglu, Serife Ciloglu, Nur Kir, Belgin Izgi

**Affiliations:** Istanbul Faculty of Medicine, Department of Ophthalmology, Istanbul University, 34093 Istanbul, Turkey

## Abstract

*Purpose*. To investigate peripapillary retinoschisis and its effect on retinal nerve fiber layer (RNFL) thickness measurements by using spectral-domain optical coherence tomography (SD-OCT) in glaucomatous eyes.* Methods*. Circumpapillary RNFL (cpRNFL) B-scan images of 940 glaucoma patients (Group 1) and 801 glaucoma-suspect patients (Group 2) obtained by SD-OCT were reviewed. The structural and clinical characteristics of the retinoschisis were investigated. The RNFL thickness measurements taken at the time of retinoschisis diagnosis and at the follow-up visits were also compared.* Results*. Twenty-nine retinoschisis areas were found in 26 of the 940 glaucoma patients (3.1%) in Group 1 and seven areas were found in 801 patients (0.87%) in Group 2. In glaucomatous eyes, the retinoschisis was attached to the optic disc and overlapped with the RNFL defect. At the time of retinoschisis, the RNFL thickness was statistically greater in the inferior temporal quadrant when compared with the follow-up scans (*p* < 0.001). No macular involvement or retinal detachment was observed.* Conclusion*. The present study investigated 33 peripapillary retinoschisis patients. Increase in RNFL thickness measurements was observed at the time of retinoschisis. It is important to examine the cpRNFL B-scan images of glaucoma patients so that the RNFL thickness is not overestimated.

## 1. Introduction

Macular and peripapillary retinoschisis has been described in X-linked retinoschisis, degenerative myopia, and congenital optic disc abnormalities such as optic pit, optic disc coloboma, tilted disc syndrome, and Morning Glory syndrome [1–7]. Peripapillary retinoschisis is characterized by the splitting of the peripapillary retinal nerve fiber layer. Previously, several case reports have described peripapillary retinoschisis in patients with different types of glaucoma, including primary open angle glaucoma, normal-tension glaucoma, narrow angle glaucoma, angle-closure glaucoma, and pseudoexfoliation glaucoma [8–12]. However, the clinical features were variable and the pathogenic mechanisms were different.

Optical coherence tomography (OCT) is a high-resolution cross-sectional imaging technique that allows for* in vivo* quantification of the retinal nerve fiber layer (RNFL) and the optic disc features, thereby enabling determination of both the presence and progression of glaucomatous structural damage [13, 14]. The measurement of RNFL thickness differences in follow-up visits is very important, especially for detecting the progression of glaucoma.

More recently, two different studies with larger samples of peripapillary retinoschisis in glaucomatous eyes have been published [15, 16]. One study was about the structural and clinical characteristics [15] and the other was about the effect of retinoschisis on RNFL thickness [16]. In this study, we wanted to investigate both the structural and clinical characteristics of peripapillary retinoschisis and examine the effects which those two factors had on RNFL thickness measurement by using spectral-domain optical coherence tomography (SD-OCT) in glaucomatous eyes.

## 2. Methods

### 2.1. Ethics Statement

The study protocol was approved by the Ethics Committee of Istanbul University, Istanbul Faculty of Medicine. The research follows the tenets of the Declaration of Helsinki. Informed consent was obtained from all participants.

### 2.2. Study Subjects

This is a cross-sectional study done at Istanbul University, Istanbul Faculty of Medicine, Ophthalmology Department, Glaucoma Unit. We reviewed the circumpapillary RNFL (cpRNFL) B-scan images of 940 glaucoma patients (Group 1) and 801 glaucoma-suspect patients (Group 2) obtained by SD-OCT (Spectralis® OCT, Heidelberg Engineering, Heidelberg, Germany) between October 2013 and May 2014, and we evaluated the RNFL thickness measurements of both groups.

All the patients underwent a full ophthalmic examination, including assessment of best-corrected visual acuity, measurement of refractive error with a TRK-1P autorefractor keratometer (Topcon, Tokyo, Japan), slit-lamp biomicroscopy, measurement of intraocular pressure (IOP) with a Goldmann applanation tonometer, and examination of the optic nerve head (ONH) and fundus with a 90-dioptre lens and a 78-dioptre lens. All patients also underwent central corneal thickness measurement using an ultrasonic pachymeter (OcuScan*™*, Alcon Inc., Irvine, CA, USA), red-free fundus photography, infrared (IR) fundus photography, and a 30-2 Swedish Interactive Threshold Algorithm standard automated visual field (VF) test (Humphrey Field Analyzer II 750, Carl Zeiss Meditec, Dublin, CA, USA).

The SD-OCT examination included IR imaging of the optic disc and peripapillary area, a cpRNFL scan, and a macular scan. Only well-centered images with a signal strength >20 db were used for the analysis.

The RNFL thickness was measured around the disc with consecutive circular B-scans (3.5 mm diameter, 768 A-scans); an online tracking system was used to compensate for eye movement. The RNFL thickness (from the inner margin of the internal limiting membrane to the outer margin of the RNFL layer) was automatically segmented using Spectralis software version 5.8.3.0.

We defined peripapillary retinoschisis as the splitting of the peripapillary RNFL with the presence of schisis cavities within the RNFL adjoining the optic disc margin in the cpRNFL SD-OCT B-scans. The circular extension of the retinoschisis was determined based on the IR imaging and cpRNFL B-scans.

Glaucoma was defined as the presence of glaucomatous optic nerve damage; neuroretinal rim notching or thinning; or RNFL defect and associated visual field defects. A glaucomatous visual field defect was defined as a visual field that was outside the normal limits on the glaucoma hemifield test or three abnormal points with *p* < 5% of being normal, one with *p* < 1% by pattern deviation, or a pattern standard deviation of <5% if the visual field was otherwise normal, as confirmed by at least two consecutive tests. The glaucoma-suspect patients had intraocular pressure (IOP) higher than ≥ 21 mmHg or a suspicious optic nerve head with normal RNFL thickness.

Among the eyes with glaucoma, those with peripapillary retinoschisis were assigned to the retinoschisis group (Group A) and, among the glaucoma-suspect patients, eyes with peripapillary retinoschisis were assigned to the RNFL thickness normal group (Group B).

The exclusion criteria for both the glaucoma and glaucoma-suspect patients were eyes with visual acuity of <8/20, a spherical equivalent refraction of $\begin{array}{c} <-12.0 \end{array}$ or >+3.0 dioptres, and eyes having concurrent retinal disease (e.g., vascular disorder or macular degeneration), congenital optic disc anomalies, or neurologic disease that could cause visual field loss.

### 2.3. Statistical Analyses

The Mann-Whitney *U* test was used to compare the clinical characteristics between the two groups, including age, spherical equivalent, and best-corrected visual acuity, IOP at SD-OCT scanning, number of antiglaucomatous medications, visual field MD, and central corneal thickness and RNFL thickness measurements at the time in which the peripapillary retinoschisis was observed. The Wilcoxon signed-rank test was used to compare the IOP and the RNFL thickness at the time of peripapillary retinoschisis and at the follow-up visits within the same eye. Statistical analyses were performed using SPSS V.16.0 (SPSS, Chicago, IL, USA).

## 3. Results

The cpRNFL B-scan images of 940 glaucoma patients and 801 glaucoma-suspect patients were reviewed. In the 940 glaucoma patients, we found 29 glaucomatous eyes with peripapillary retinoschisis in 26 patients (17 women and nine men), three of whom had bilateral peripapillary retinoschisis (Group A). In the 801 glaucoma-suspect patients, we found seven patients (four women and three men) with unilateral peripapillary retinoschisis (Group B). In Group A, seven patients had clinically unilateral pseudoexfoliation glaucoma, three had juvenile glaucoma, three had angle-closure glaucoma, and 13 had primary open angle glaucoma. In Group B, five patients were diagnosed with ocular hypertension and two were only glaucoma-suspect.

The clinical characteristics of the two groups are listed in Table 1. Age, spherical equivalent, best-corrected visual acuity, IOP at SD-OCT scanning, visual field MD, and central corneal thicknesses were not significantly different between the two groups (*p* > 0.05). Only the number of antiglaucomatous medications was significantly higher in Group A (*p* = 0.009).

For the eyes in the glaucomatous retinoschisis group, the peripapillary retinoschisis was located in the inferior temporal quadrant in seven eyes (24.13%); it was located in the inferior nasal quadrant in three eyes (10.34%), in the nasal quadrant in one eye (3.44%), in the temporal quadrant in four eyes (13.79%), in the superior nasal quadrant in three eyes (10.34%), in the superior temporal quadrant in four eyes (13.79%), and in more than one quadrant in seven eyes (24.13%). In the glaucoma-suspect retinoschisis group, the peripapillary retinoschisis was located in the superior temporal quadrant in two eyes (28.57%); it was located in the temporal quadrant in two eyes (28.57%), in the inferior temporal quadrant in one eye (14.28%), in the inferior quadrant in one eye (14.28%), and in both the nasal and nasal inferior quadrants in one eye (14.28%).

**Figure 1:**
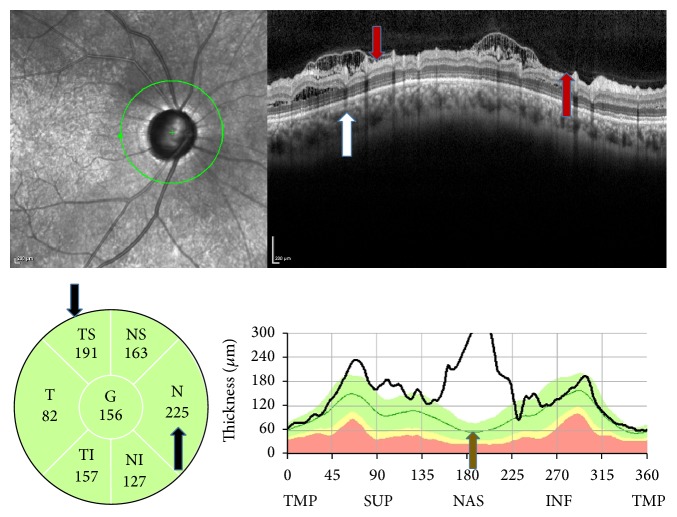
SD-OCT findings in a case of pseudoexfoliation glaucoma. The retinoschisis observed in the B-scan image (red arrows) in more than one sector and in different layers (white arrow), in the sector thickness map (black arrows), and in the TSNIT graphs (green arrow).

The involved retinal layers varied among the retinoschisis cases. In both groups, it was detected throughout the inner retinal layers, including the nerve fiber layer, the ganglion cell layer, and the inner plexiform layer (Figure 1). There was no macular involvement or retinal detachment in any of the patients and optic disc pit was not seen in any of the patients upon fundus examination.

The RNFL thickness measurements at the time of peripapillary retinoschisis in both groups are listed in Table 2. The average, superior, and inferior quadrant RNFL thickness measurements were significantly greater in Group B (*p* < 0.005). However, the RNFL thickness measurements of the nasal and temporal quadrants were not significantly different between the two groups (*p* > 0.05).

We conducted the follow-up cpRNFL measurements using SD-OCT in 19 patients in Group A after a time interval of 5.8 ± 2.02 months and in six patients in Group B after a time interval of 6.6 ± 0.8 months. In the patients in our study, retinoschisis did not resolve; however, in most of the cases, the extension of the retinoschisis area was smaller in the follow-up scans. The differences between the RNFL thickness measurements and the intraocular pressure at the time of peripapillary retinoschisis and at the time of the follow-up visits are shown in Table 3. Only the RNFL thickness measurement of the inferior temporal quadrant was significantly greater at the time of peripapillary retinoschisis than at the follow-up visit (*p* = 0.008) in Group A. There was no significant difference in the other quadrants in both groups.

## 4. Discussion

In this study, we reviewed cpRNFL B-scan images of a total of 1741 patients (940 glaucoma patients and 801 glaucoma-suspect patients) obtained by Spectralis SD-OCT. We found 29 peripapillary retinoschisis areas in glaucoma group (3.1%) and seven peripapillary retinoschisis areas in the glaucoma-suspect group (0.87%). We investigated the clinical characteristics and the effect of retinoschisis on the RNFL measurements and we aimed to determine if these were also present during follow-up of the glaucoma patients.

The prevalence and the factors associated with peripapillary retinoschisis are not well known, yet. In our study, we only investigated the patients for whom the RNFL measurements had been taken for glaucoma and the suspicion of glaucoma, so we do not have data from a healthy group. However, according to our findings retinoschisis is seen more frequently in glaucoma patients (3.1% versus 0.87%). Lee et al. investigated the structural and clinical characteristics of peripapillary retinoschisis in glaucoma patients [15]. They found 25 cases of peripapillary retinoschisis in 372 open angle glaucoma patients (5.9%) and only one in 187 healthy control subjects (0.5%). The difference between their findings and ours may be due to the heterogeneity of the glaucoma patients in our study and the sample size. In our glaucoma group, 13 (50%) patients had POAG, seven (26.9%) patients had clinically unilateral pseudoexfoliation glaucoma, three (11.5%) patients had juvenile glaucoma, and three (11.5%) patients had angle-closure glaucoma. According to their data and our data, we can say that peripapillary retinoschisis is seen more frequently in open angle glaucoma.

In the present study population, peripapillary retinoschisis was most commonly found in the inferior temporal quadrant, followed by the superior temporal quadrant and the temporal quadrant. Hwang et al. investigated 21 open angle glaucoma patients with peripapillary retinoschisis and compared them with 38 glaucoma patients without peripapillary retinoschisis [16]. In their study, peripapillary retinoschisis was most commonly found in the superior quadrant, followed by the inferior and the nasal quadrants. Therefore, we can say that peripapillary retinoschisis can be seen in every quadrant and it can also be seen in more than one quadrant.

In the population studied by Hwang et al., all of the peripapillary retinoschisis cases were transient and they were resolved without additional management within a median period of nine months [16]. Lee et al. followed 13 patients for more than one year and the retinoschisis resolved in seven of the patients [15]. Kahook et al. described two cases of peripapillary retinoschisis and macular retinoschisis with narrow angles and increased intraocular pressure [11]. The retinoschisis resolved in one eye of one case while it appeared in the fellow eye. Farjad et al. described a patient who developed peripapillary retinoschisis associated with an underlying serous detachment in an eye with large optic nerve cupping secondary to primary open angle glaucoma [8]. In their patient, the retinoschisis resolved spontaneously. In our study population, we followed up 19 patients in Group A and six patients in Group B for a median time of seven months. In our study, retinoschisis did not resolve; however, in most of the patients the extension of the retinoschisis area was smaller in the follow-up scans.

The occurrence of macular schisis and serous detachment isolated to glaucoma patients has been documented in several reports [10, 11, 17]. Hollander et al. reported the development of macular schisis in a patient with intermittent angle-closure glaucoma [10]. Kahook et al. also reported two cases of peripapillary retinoschisis and macular retinoschisis with narrow angles and increased intraocular pressure [11]. Zumbro et al. documented macular schisis with detachment in five glaucoma patients with enlarged cupping without an optic nerve pit [17]. Lee et al. described 25 cases of peripapillary retinoschisis in POAG patients, but there was no macular involvement [15]. Hwang et al. also documented 21 cases of peripapillary retinoschisis in 19 POAG patients without macular involvement [16]. In our study, we also did not observe any macular involvement or retinal detachment in our patients. Therefore, we suggest that macular involvement by the expansion of retinoschisis is not a common finding when the retinoschisis developed in the peripapillary region in glaucomatous eyes.

Fluctuation or elevation of IOP is one of the possible causes of peripapillary retinoschisis in glaucoma [10, 11, 17]. In the patients in the study conducted by Lee et al., IOP at the time of OCT scan was significantly associated in both univariate and multivariate analyses [15]. However, in six cases they found retinoschisis during the follow-up without IOP elevation. In the study conducted by Hwang et al., all the eyes had stable IOP with medical treatment and the eyes with peripapillary retinoschisis did not have greater IOP fluctuation than the eyes without peripapillary retinoschisis [16]. In our study, all the patients in Group A had stable IOP with medical treatment. However, the possibility of sustained, cumulative IOP-induced stress, and IOP elevation or IOP fluctuation that was not detected at the clinic cannot be ruled out. Zumbro et al. reported a case in which the retinoschisis resolved after filtering surgery [17]. In our cases, retinoschisis did not resolve but the extension area of the retinoschisis decreased in the follow-up scans. However, no statistically significant difference was found between the IOP levels. Therefore, further studies involving large samples and a longer follow-up time are needed to investigate the relationship between IOP fluctuation and the development and resolution of the retinoschisis.

**Figure 2:**
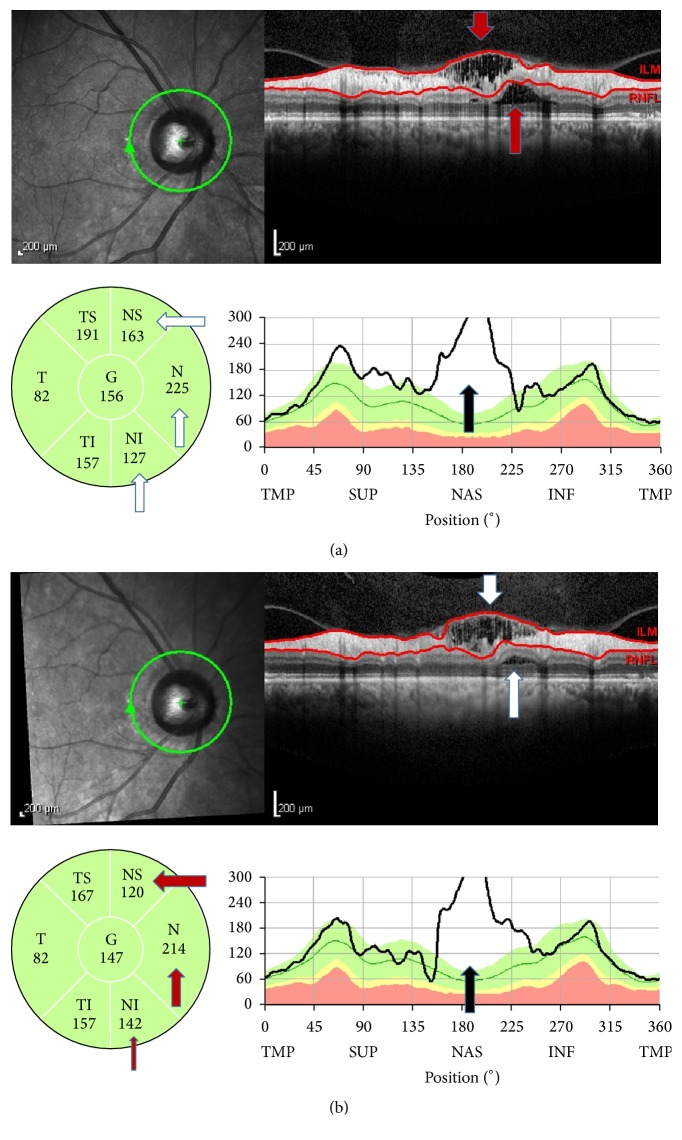
SD-OCT findings in a case of glaucoma-suspect. She had a large disc with cupping but IOP was under 15 mmHg. (a) The retinoschisis observed in the B-scan image (red arrows), in the sector thickness map (white arrows), and in the TSNIT graphs (black arrow). (b) The extension of retinoschisis was smaller 3 months later in B-scan image (white arrows), in the sector thickness map (red arrows), and in the TSNIT graphs (black arrow).

In the present study, the retinoschisis was attached to the optic disc and overlapped with the RNFL defect according to the horizontal B-scans in glaucomatous patients. This finding was identical to the results found in the series conducted by Lee et al. and Hwang et al. [15, 16]. In the Lee et al. study, acquired optic nerve pit was found in eight patients; it was not found in the present study and in the series conducted by Hwang et al. Optic nerve pit was also not seen in the cases reported in other studies [8, 10, 11]. Those investigators suggested that sustained elevation of IOP and optic nerve cupping may have produced a direct communication route, like microscopic interconnections, to the adjacent retina that enabled vitreous fluid migration. Lee et al. also speculated that the damage to the lamina cribrosa may provide a conduit that allows fluid to travel from either the vitreous cavity or the subarachnoid space [15, 18–20]. In our study population, in addition to the glaucomatous patients, two patients in the glaucoma suspected group had optic nerve cupping with IOP under 15 mmHg. Thus, optic nerve damage alone is also a risk factor in the development of retinoschisis (Figure 2).

**Figure 3:**
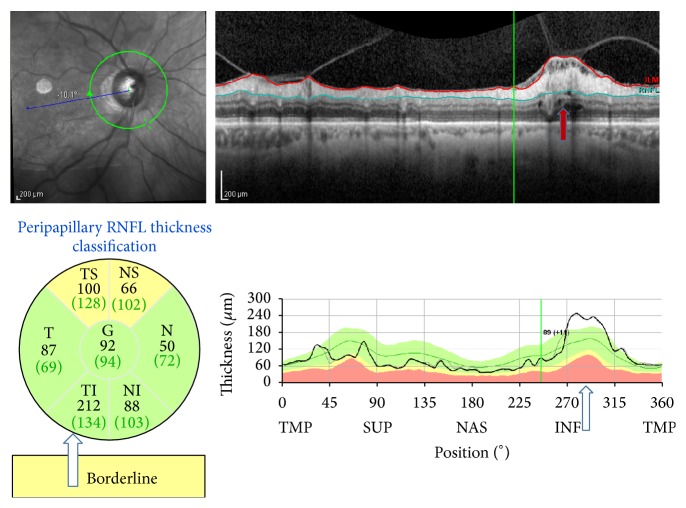
SD-OCT findings in a case of primary open angle glaucoma. The retinoschisis observed with vitreopapillary traction in the B-scan image (red arrows). The remarkable increase in the RNFL thickness in the inferotemporal area is also seen in the TSNIT graphs (white arrows).

Vitreopapillary traction is another possible mechanism of peripapillary retinoschisis. Batta et al. and Hwang et al. reported that the RNFL thickness measured by OCT was greater at the point of vitreous attachment and vitreopapillary traction [21, 22]. Studies have suggested that vitreous traction may facilitate the entering of liquefied vitreous fluid into the retina. In those studies, vitreous traction was not documented with peripapillary retinoschisis; however, in one of our pseudoexfoliation glaucoma patients, we found retinoschisis with vitreous traction (Figure 3) and, consequently, the RNFL thickness was greater in that area.

**Figure 4:**
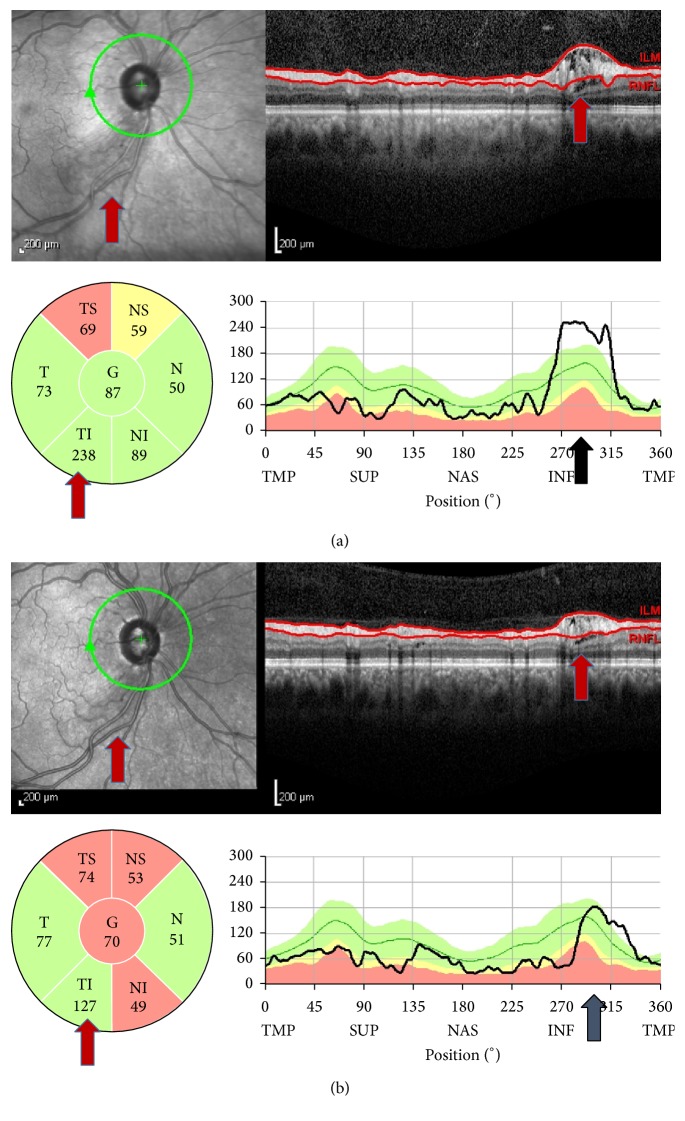
SD-OCT findings in a case of juvenile glaucoma. (a) The retinoschisis observed in the B-scan image (red arrows). (b) The extension of retinoschisis was smaller 3 months later (red arrows). According to the sector thickness map, inferotemporal RNFL was decreased from 238 *μ*m to 127 *μ*m. The remarkable decrease in the RNFL thickness in the inferotemporal area is also seen in the TSNIT graphs (black arrows).

Transient increase in the RNFL thickness measurements is found in eyes with peripapillary retinoschisis and, after the resolution of retinoschisis, the RNFL thickness was found to decrease remarkably. If a clinician simply looks at the measurement data without noticing the retinoschisis in the horizontal B-scans or the IR images, he or she would consider such a decrease to be a rapid progression of the retinoschisis; or if it was the first scan, he or she might overestimate the RNFL as being falsely thick (Figure 4). We propose that clinicians should examine the thickness maps as well as the horizontal B-scans in order to rule out retinoschisis so as not to overestimate the RNFL thickness or misinterpret the resolution of retinoschisis as a rapid structural progression.

In conclusion, peripapillary retinoschisis was observed in glaucomatous eyes and it affected the RNFL thickness measurement obtained by Spectralis SD-OCT. Further studies with large samples and a longer follow-up time are needed to determine the pathogenic mechanism and its correlation with glaucoma.

## Figures and Tables

**Table 1:** Clinical characteristics of the patients.

	Group A (*n* = 29)	Group B (*n* = 7)	*Z*-value^*∗*^	*p* value^*∗*^
Age (years)	63.45 ± 13.5	64.57 ± 8.03	−0.306	0.780
Spherical equivalent (dioptre)	−2.47 ± 4.2	−0.78 ± 1.8	−0.439	0.668
Best-corrected visual acuity (BCVA)	0.79 ± 0.22	0.91 ± 0.12	−0.440	0.695
IOP at SD-OCT scanning (mmHg)	15.55 ± 4.16	16.71 ± 4.82	−1.052	0.302
Number of antiglaucomatous medication	2.48 ± 1.18	0.71 ± 0.95	−2.625	**0.009**
Visual field MD (dB)	−8.22 ± 7.08	−3.8 ± 2.5	−1.457	0.160
Central corneal thickness (*μ*m)	542.75 ± 41.69	526.57 ± 34.9	−0.495	0.641

^**∗**^Mann-Whitney *U* test.

**Table 2:** RNFL thickness measurements at the time of peripapillary retinoschisis.

	Group A (*n* = 29)	Group B (*n* = 7)	*Z*-value^*∗*^	*p* value^*∗*^
Average	78.86 ± 22.07	119.57 ± 26.12	−3,140	**0,001**
*Sectors*				
Superior nasal	77.03 ± 41.98	127.42 ± 27.22	−2,247	**0,024**
Superior temporal	86.34 ± 30.99	166.85 ± 73.49	−2,855	**0,03**
Nasal	66.27 ± 50.13	94.28 ± 59.32	−1,789	0,077
Temporal	70.44 ± 16.64	103.85 ± 49.29	−,990	0,339
Inferior nasal	81.41 ± 35.02	115.57 ± 14.55	−2,855	**0,003**
Inferior temporal	111.86 ± 52.0	150.28 ± 14.26	−2,740	**0,005**

^**∗**^Mann-Whitney *U* test.

**Table 3:** RNFL thickness changes in retinoschisis patients.

	Group A (*n* = 19)		*p* value^*∗*^	Group B (*n* = 6)		*p* value^*∗*^
	First visit	Follow-up		First visit	Follow-up	
IOP (mmHg)	15.3 ± 3.4	15.7 ± 3.5	0.917	17.5 ± 4.8	15 ± 1.3	0.698
*RNFL thickness (µm)*						
Average	74.16 ± 17.5	72.84 ± 16.6	0.498	123.3 ± 26.5	128.5 ± 18.94	0.105
*Sectors*						
Superior nasal	63 ± 30.4	64.78 ± 25.2	0.735	129 ± 29.5	125.5 ± 30.9	0.909
Superior temporal	82.36 ± 26.9	93.58 ± 40.7	0.866	173.3 ± 78.3	166.18 ± 64.8	0.198
Nasal	51.79 ± 35.3	47.78 ± 23.8	0.866	99 ± 63.5	113.83 ± 64.9	0.698
Temporal	71.57 ± 17.9	69.47 ± 20.9	0.463	110 ± 51	116.3 ± 61.7	0.442
Inferior nasal	82.2 ± 30.9	86.05 ± 33.9	1.000	116.3 ± 15.8	119.7 ± 28.1	1.000
Inferior temporal	121.11 ± 52.7	103.63 ± 42.7	**0.008**	150.17 ± 15.6	155.3 ± 26.9	0.917

^*∗*^Wilcoxon signed-rank test.

## References

[1] George N. D. L., Yates J. R. W., Moore A. T. (1996). Clinical features in affected males with X-linked retinoschisis. *Archives of Ophthalmology*.

[2] Rosenfeld P. J., Flynn H. W., McDonald H. R. (1998). Outcomes of vitreoretinal surgery in patients with X-linked retinoschisis. *Ophthalmic Surgery and Lasers*.

[3] Hirakata A., Hida T., Ogasawara A., Iizuka N. (2005). Multilayered retinoschisis associated with optic disc pit. *Japanese Journal of Ophthalmology*.

[4] Scott I. U., Moshfeghi A. A., Flynn H. W. (2006). Surgical management of macular retinoschisis associated with high myopia. *Archives of Ophthalmology*.

[5] Tang J., Rivers M. B., Moshfeghi A. A., Flynn H. W., Chan C.-C. (2010). Pathology of macular foveoschisis associated with degenerative myopia. *Journal of Ophthalmology*.

[6] Miura G., Yamamoto S., Tojo N., Mizunoya S. (2006). Foveal retinal detachment and retinoschisis without macular hole associated with tilted disc syndrome. *Japanese Journal of Ophthalmology*.

[7] Chang S., Gregory-Roberts E., Chen R. (2012). Retinal detachment associated with optic disc colobomas and morning glory syndrome. *Eye*.

[8] Farjad H., Besada E., Frauens B. J. (2010). Peripapillary schisis with serous detachment in advanced glaucoma. *Optometry and Vision Science*.

[9] Zhao M., Li X. (2011). Macular retinoschisis associated with normal tension glaucoma. *Graefe's Archive for Clinical and Experimental Ophthalmology*.

[10] Hollander D. A., Barricks M. E., Duncan J. L., Irvine A. R. (2005). Macular schisis detachment associated with angle-closure glaucoma. *Archives of Ophthalmology*.

[11] Kahook M. Y., Noecker R. J., Ishikawa H. (2007). Peripapillary schisis in glaucoma patients with narrow angles and increased intraocular pressure. *American Journal of Ophthalmology*.

[12] Ornek N., Büyüktortop N., Ornek K. (2013). Peripapillary and macular retinoschisis in a patient with pseudoexfoliation glaucoma. *BMJ Case Reports*.

[13] Greenfield D. S., Weinreb R. N. (2008). Role of optic nerve imaging in glaucoma clinical practice and clinical trials. *American Journal of Ophthalmology*.

[14] Suh M. H., Yoo B. W., Park K. H., Kim H., Kim H. C. (2015). Reproducibility of spectral-domain optical coherence tomography RNFL map for glaucomatous and fellow normal eyes in unilateral glaucoma. *Journal of Glaucoma*.

[15] Lee E. J., Kim T.-W., Kim M., Choi Y. J. (2014). Peripapillary retinoschisis in glaucomatous eyes. *PLoS ONE*.

[16] Hwang Y. H., Kim Y. Y., Kim H. K., Sohn Y. H. (2014). Effect of peripapillary retinoschisis on retinal nerve fibre layer thickness measurement in glaucomatous eyes. *British Journal of Ophthalmology*.

[17] Zumbro D. S., Jampol L. M., Folk J. C., Olivier M. M. G., Anderson-Nelson S. (2007). Macular schisis and detachment associated with presumed acquired enlarged optic nerve head cups. *American Journal of Ophthalmology*.

[18] Georgalas I., Ladas I., Georgopoulos G., Petrou P. (2011). Optic disc pit: a review. *Graefe's Archive for Clinical and Experimental Ophthalmology*.

[19] Krivoy D., Gentile R., Liebmann J. M., Stegman Z., Walsh J. B., Ritch R. (1996). Imaging congenital optic disc pits and associated maculopathy using optical coherence tomography. *Archives of Ophthalmology*.

[20] Kuhn F., Kover F., Szabo I., Mester V. (2006). Intracranial migration of silicone oil from an eye with optic pit. *Graefe's Archive for Clinical and Experimental Ophthalmology*.

[21] Batta P., Engel H. M., Shrivastava A., Freeman K., Mian U. (2010). Effect of partial posterior vitreous detachment on retinal nerve fiber layer thickness as measured by optical coherence tomography. *Archives of Ophthalmology*.

[22] Hwang Y. H., Kim Y. Y. (2015). Peripapillary retinal nerve fiber layer thickening associated with vitreopapillary traction. *Seminars in Ophthalmology*.

